# Supplementary material for: Drug-resistant TB prevalence study in 5 health institutions in Haiti
Source: PLoS One. 2021 Mar 18;16(3):e0248707. doi: 10.1371/journal.pone.0248707 (PMC7971505; doi:10.1371/journal.pone.0248707)
Supplement: S1 Fig — Total of 74 DR-TB strains including 12 mono-drug resistant strains and 62 multi-drug resistant TB strains. GHESKIO (INLR): n = 34. IMIS: n = 34. HUM: n = 3. HUJ: n = 2. HIC: n = 1. I: Isoniazid. R: Rifampin. S: Streptomycin. E: Ethambutol. PZA: Pyrazinamide. KM: Kanamycin. (DOCX) [file pone.0248707.s001.docx]

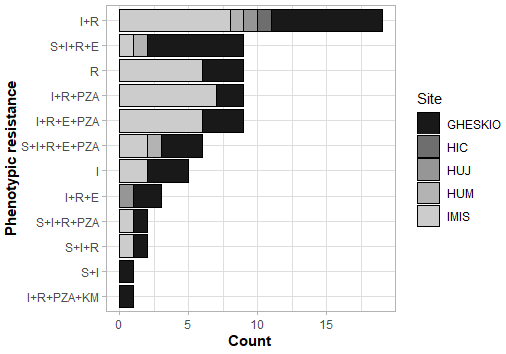


### **Figure 1S. Description of DR-TB strains phenotypes among all patients with available DST results.** Total of 74 DR-TB strains including 12 mono-drug resistant strains and 62 multi-drug resistant TB strains. GHESKIO (INLR): n = 34. IMIS: n = 34. HUM: n = 3. HUJ: n = 2. HIC: n = 1. I: Isoniazid. R: Rifampin. S: Streptomycin. E: Ethambutol. PZA: Pyrazinamide. KM: Kanamycin.
