# Supplementary figures and images for: Drug-resistant TB prevalence study in 5 health institutions in Haiti
Source: PLoS One. 2021 Mar 18;16(3):e0248707. doi: 10.1371/journal.pone.0248707 (PMC7971505; doi:10.1371/journal.pone.0248707)

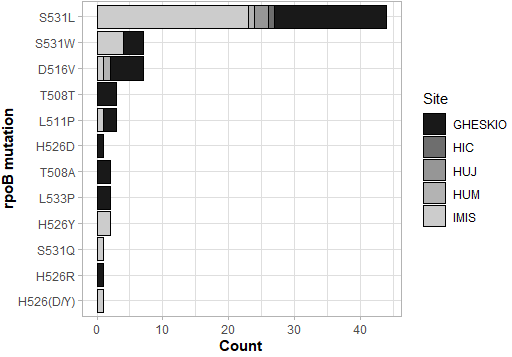


### Figure 2S. *rpoB* genotypic diversity of DR-TB isolates (n = 74).

Supplement: S2 Fig — (DOCX) [file pone.0248707.s002.docx]
