# Supplementary material for: Drug-resistant TB prevalence study in 5 health institutions in Haiti
Source: PLoS One. 2021 Mar 18;16(3):e0248707. doi: 10.1371/journal.pone.0248707 (PMC7971505; doi:10.1371/journal.pone.0248707)
Supplement: S3 Fig — Data are given for all patients with known spoligotypes. (DOCX) [file pone.0248707.s003.docx]

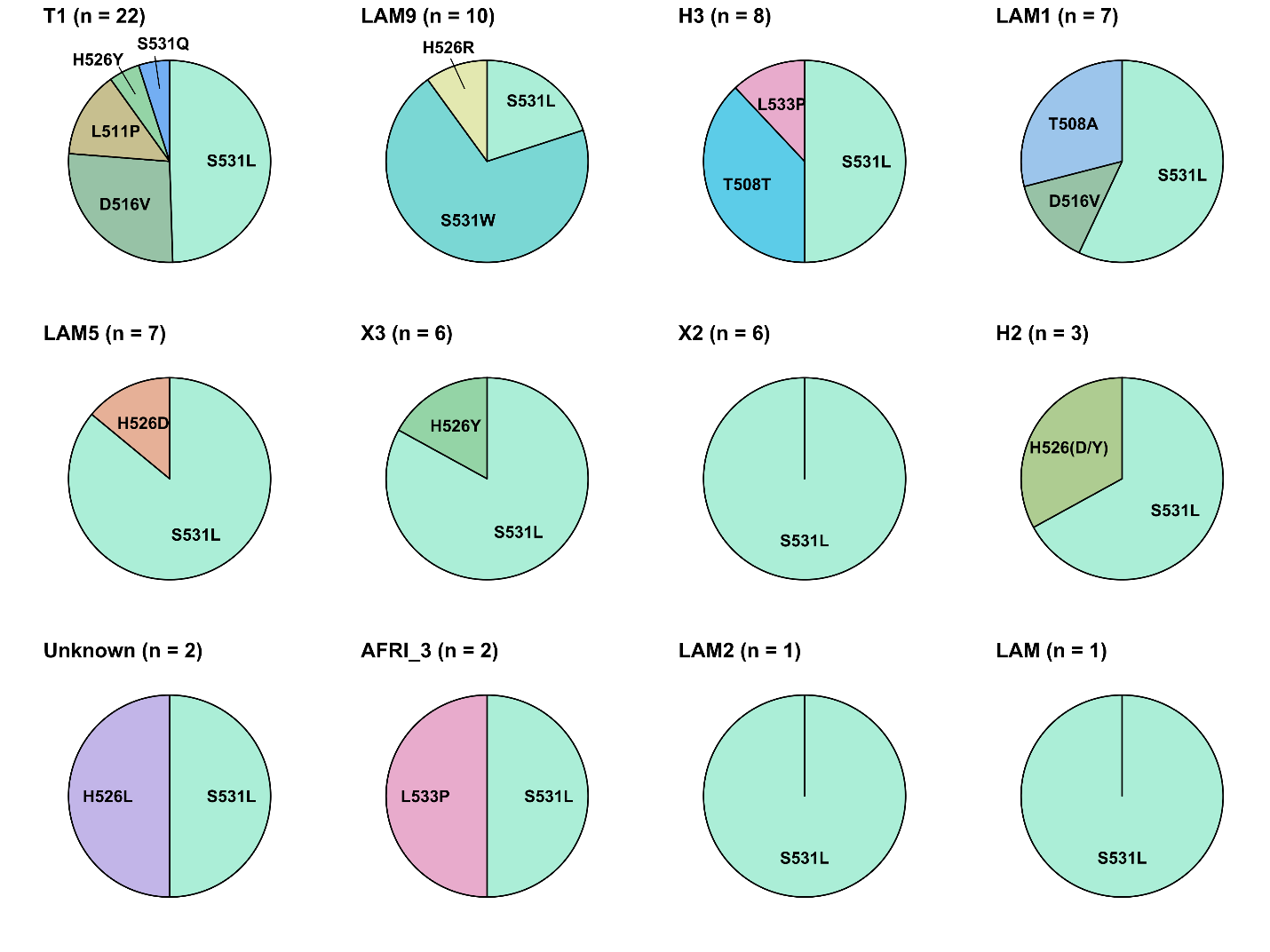


### Figure 3S. Frequency of detected rpoB mutation in each identified drug-resistant *M. tuberculosis* lineage (n=74). Data are given for all patients with known spoligotypes.
