# Supplementary material for: Drug-resistant TB prevalence study in 5 health institutions in Haiti
Source: PLoS One. 2021 Mar 18;16(3):e0248707. doi: 10.1371/journal.pone.0248707 (PMC7971505; doi:10.1371/journal.pone.0248707)
Supplement: S1 Table — (DOCX) [file pone.0248707.s004.docx]

### Table 1S : Summary of resistance profiles of DR-TB isolates identified in new TB cases, relapse, treatment after failure or treatment after interruption.

| **Resistance profiles** | **New cases  *N* (%)** | **Relapse  *N* (%)** | **Treatment after failure  *N* (%)** | **Treatment after interruption  *N* (%)** | ***P*** | **Total  N (%)** |
| --- | --- | --- | --- | --- | --- | --- |
|  | N_T_=2,401 | N_T_=236 | N_T_=30 | N_T_=110 |  | N_T_=2,777 |
| Resistant to INH* | 56 (2.3) | 8 (3.4) | 1 (3.3) | - | *0.148* | 65 (2.3) |
| Resistant to RIF* | 57 (2.4) | 10 (4.2) | 1 (3.3) | - | *0.053* | 68 (2.4) |
| Resistant to EMB* | 24 (1.0) | 2 (0.8) | 1 (3.3) | - | *1.000* | 27 (1.0) |
| Resistant to STR* | 15 (0.6) | 3 (1.3) | - | - | *0.431* | 18 (0.6) |
| Resistant to ETH* | 11 (0.5) | 1 (0.4) | - | - | *0.846* | 12 (0.4) |
| Resistant to PAS* | 1 (0.0) | - | - | - | *1.000* | 1 (0.0) |
| Resistant to PZA* | 23 (1.0) | 2 (0.8) | - | - | *0.720* | 25 (0.9) |
| Monoresistant to INH | 2 (0.1) | 1 (0.4) | - | - | *0.448* | 3 (0.1) |
| Monoresistant to RIF | 6 (0.2) | 3 (1.3) | - | - | *0.084* | 9 (0.3) |
| Monoresistant to EMB | - | - | - | - | *1.000* | - |
| Monoresistant to STR | - | - | - | - | *1.000* | - |
| Monoresistant to ETH | - | - | - | - | *1.000* | - |
| Monoresistant to PAS | - | - | - | - | *1.000* | - |
| Monoresistant to PZA | - | - | - | - | *1.000* | - |
| Resistant to 1 AB** | 8 (0.3) | 4 (1.7) | - | - | *0.067* | 12 (0.4) |
| Resistant to 2 AB** | 18 (0.7) | 2 (0.8) | - | - | *0.893* | 20 (0.7) |
| Resistant to 3 AB** | 11 (0.5) | 3 (1.3) | 1 (3.3) | - | *0.172* | 15 (0.5) |
| Resistant to 4 AB** | 13 (0.5) | 1 (0.4) | - | - | *1.000* | 14 (0.5) |
| Resistant to 5 AB** | 12 (0.5) | 1 (0.4) | - | - | *1.000* | 13 (0.5) |

***Footnotes*** *: *comprises all drug-resistant isolates to the respective drugs (irrespective of other associated resistances). INH (isoniazid), RIF (Rifampin), EMB (ethambutol), STR (streptomycin), ETH (ethionamide), PAS (para-aminosalicylic acid) or PZA (Pyrazinamide), respectively.** Strains resistant to 1, 2, 3 or 4 of the tested antibiotics (AB)*
