# Supplementary material for: Drug-resistant TB prevalence study in 5 health institutions in Haiti
Source: PLoS One. 2021 Mar 18;16(3):e0248707. doi: 10.1371/journal.pone.0248707 (PMC7971505; doi:10.1371/journal.pone.0248707)
Supplement: S2 Table — (DOCX) [file pone.0248707.s005.docx]

### Table 2S: Mono-resistance profile of DR-TB isolates identified in new TB cases, relapse, treatment after failure, treatment after interruption.

| **TB strain resistance profile** | **Antibiotic resistance profile** | **New cases** | |  | **Relapse** | |  | **Treatment after failure** | |  | **Treatment after interruption** | |  | **TOTAL** | |
| --- | --- | --- | --- | --- | --- | --- | --- | --- | --- | --- | --- | --- | --- | --- | --- |
|  |  | N | *%* |  | N | *%* |  | N | *%* |  | N | *%* |  | N | *%* |
| Mono-drug resistant TB | INH | 2 | *25* |  | 1 | *25* |  | 0 | *-* |  | 0 | *-* |  | 3 | *25* |
|  | RIF | 6 | *75* |  | 3 | *75* |  | 0 | *-* |  | 0 | *-* |  | 9 | *75* |
|  | STR | 0 | *0* |  | 0 | *0* |  | 0 | *-* |  | 0 | *-* |  | 0 | *0* |
|  | EMB | 0 | *0* |  | 0 | *0* |  | 0 | *-* |  | 0 | *-* |  | 0 | *0* |
|  | PZA | 0 | *0* |  | 0 | *0* |  | 0 | *-* |  | 0 | *-* |  | 0 | *0* |
| Total mono-drug resistant isolates | | **8** | *100* |  | **4** | *100* |  | **0** | *-* |  | **0** | *-* |  | **12** | *100* |
