# Supplementary material for: Drug-resistant TB prevalence study in 5 health institutions in Haiti
Source: PLoS One. 2021 Mar 18;16(3):e0248707. doi: 10.1371/journal.pone.0248707 (PMC7971505; doi:10.1371/journal.pone.0248707)
Supplement: S3 Table — (DOCX) [file pone.0248707.s006.docx]

### Table 3S: Multi-resistant profile of DR-TB isolates identified in new TB cases, relapse, treatment after failure, treatment after interruption.

|  |  | **New cases** | |  | **Relapse** | |  | **Treatment after failure** | |  | **Treatment after interruption** | |  | **TOTAL** | |
| --- | --- | --- | --- | --- | --- | --- | --- | --- | --- | --- | --- | --- | --- | --- | --- |
|  |  | N | *%* |  | N | *%* |  | N | *%* |  | N | *%* |  | N | *%* |
| Multi-drug resistant TB | INH + RIF | 16 | *29.6* |  | 2 | *28.6* |  | 0 | *0.0* |  | 0 | *-* |  | 18 | *29.0* |
|  | INH + RIF + STR | 0 | *0.0* |  | 2 | *28.6* |  | 0 | *0.0* |  | 0 | *-* |  | 2 | *3.2* |
|  | INH + RIF + EMB | 2 | *3.7* |  | 0 | *0.0* |  | 1 | *100.0* |  | 0 | *-* |  | 3 | *4.8* |
|  | INH + RIF + EMB + PZA | 5 | *9.3* |  | 1 | *14.3* |  | 0 | *0.0* |  | 0 | *-* |  | 6 | *9.7* |
|  | INH + RIF + EMB + PZA + ETH | 3 | *5.6* |  | 0 | *0.0* |  | 0 | *0.0* |  | 0 | *-* |  | 3 | *4.8* |
|  | INH + RIF + PZA | 6 | *11.1* |  | 1 | *14.3* |  | 0 | *0.0* |  | 0 | *-* |  | 7 | *11.3* |
|  | INH + RIF + ETH | 2 | *3.7* |  | 0 | *0.0* |  | 0 | *0.0* |  | 0 | *-* |  | 2 | *3.2* |
|  | INH + RIF + STR + EMB | 5 | *9.3* |  | 0 | *0.0* |  | 0 | *0.0* |  | 0 | *-* |  | 5 | *8.1* |
|  | INH + RIF + STR + EMB + ETH | 3 | *5.6* |  | 1 | *14.3* |  | 0 | *0.0* |  | 0 | *-* |  | 4 | *6.5* |
|  | INH + RIF + PZA + KM | 1 | *1.9* |  | 0 | *0.0* |  | 0 | *0.0* |  | 0 | *-* |  | 1 | *1.6* |
|  | INH + RIF + PZA + ETH | 2 | *3.7* |  | 0 | *0.0* |  | 0 | *0.0* |  | 0 | *-* |  | 2 | *3.2* |
|  | INH + RIF + STR + EMB + PZA | 6 | *11.1* |  | 0 | *0.0* |  | 0 | *0.0* |  | 0 | *-* |  | 6 | *9.7* |
|  | INH + ETH + PAS | 1 | *1.9* |  | 0 | *0.0* |  | 0 | *0.0* |  | 0 | *-* |  | 1 | *1.6* |
|  | ETH + INH | 1 | *1.9* |  | 0 | *0.0* |  | 0 | *0.0* |  | 0 | *-* |  | 1 | *1.6* |
|  | STR + INH | 1 | *1.9* |  | 0 | *0.0* |  | 0 | *0.0* |  | 0 | *-* |  | 1 | *1.6* |
| Total multi-drug-resistant isolates | | **54** | *100.0* |  | **7** | *100.0* |  | **1** | *100.0* |  | **0** | *-* |  | **62** | *100.0* |
