# Supplementary material for: Drug-resistant TB prevalence study in 5 health institutions in Haiti
Source: PLoS One. 2021 Mar 18;16(3):e0248707. doi: 10.1371/journal.pone.0248707 (PMC7971505; doi:10.1371/journal.pone.0248707)
Supplement: S4 Table — (DOCX) [file pone.0248707.s007.docx]

### Table 4S: Summary of resistance profiles of DR-TB isolates identified in new TB cases, relapse, treatment after failure, treatment after interruption.

|  |  | **New cases** | |  | **Relapse** | |  | **Treatment after failure** | |  | **Treatment after interruption** | |  | **TOTAL** | |
| --- | --- | --- | --- | --- | --- | --- | --- | --- | --- | --- | --- | --- | --- | --- | --- |
|  |  | N | *%* |  | N | *%* |  | N | *%* |  | N | *%* |  | N | *%* |
| Mono-drug resistant TB | | 8 | *12.9* |  | 4 | *36.4* |  | 0 | *0.0* |  | 0 | *-* |  | 12 | *16.2* |
| Multi-drug resistant TB | | 54 | *87.1* |  | 7 | *63.6* |  | 1 | *100.0* |  | 0 | *-* |  | 62 | *83.8* |
| Total drug-resistant isolates | | **62** | *100.0* |  | **11** | *100.0* |  | **1** | *100.0* |  | 0 | *-* |  | **74** | *100.0* |
