# Supplementary material for: Drug-resistant TB prevalence study in 5 health institutions in Haiti
Source: PLoS One. 2021 Mar 18;16(3):e0248707. doi: 10.1371/journal.pone.0248707 (PMC7971505; doi:10.1371/journal.pone.0248707)
Supplement: S5 Table — (DOCX) [file pone.0248707.s008.docx]

### Table 5S: Distribution of DR-TB lineages (SIT) among new TB cases, relapse and treatment after failure.

| **SIT** | **New cases**  **N (%)** | **Relapse**  **N (%)** | **Treatment after failure**  **N (%)** |  |
| --- | --- | --- | --- | --- |
|  | N_T_=62 | N_T_=11 | N_T_=1 | *P* |
| 2 | 1 (1.6) | 1 (9.1) | 1 (100.0) | *<0.001* |
| 5 | 4 (6.5) | 1 (9.1) | - (-) | *0.177* |
| **6** | **1 (1.6)** | - (-) | - (-) | *0.196* |
| **7** | **2 (3.2)** | - (-) | - (-) | *0.398* |
| 17 | 1 (1.6) | - (-) | - (-) | *0.196* |
| 20 | 4 (6.5) | - (-) | - (-) | *0.818* |
| 42 | 8 (12.9) | 2 (18.2) | - (-) | *0.381* |
| 50 | 2 (3.2) | - (-) | - (-) | *0.398* |
| 51 | 2 (3.2) | - (-) | - (-) | *0.398* |
| 53 | 6 (9.7) | 1 (9.1) | - (-) | *0.947* |
| 77 | 2 (3.2) | 1 (9.1) | - (-) | *0.648* |
| 91 | 5 (8.1) | - (-) | - (-) | *0.595* |
| 93 | 5 (8.1) | 2 (18.2) | - (-) | *0.543* |
| 137 | 5 (8.1) | 1 (9.1) | - (-) | *0.950* |
| 294 | 2 (3.2) | - (-) | - (-) | *0.398* |
| **373** | **1 (1.6)** | - (-) | - (-) | *0.196* |
| 408 | 1 (1.6) | - (-) | - (-) | *0.196* |
| 455 | 2 (3.2) | 2 (18.2) | - (-) | *0.126* |
| 578 | 1 (1.6) | - (-) | - (-) | *0.196* |
| 714 | 1 (1.6) | - (-) | - (-) | *0.196* |
| **909** | **1 (1.6)** | - (-) | - (-) | *0.196* |
| **1624** | **1 (1.6)** | - (-) | - (-) | *0.196* |
| UKN | 4 (6.5) | - (-) | - (-) | *0.196* |
| New SIT detected in the present study are in bold. | | | |  |
