# Supplementary material for: Drug-resistant TB prevalence study in 5 health institutions in Haiti
Source: PLoS One. 2021 Mar 18;16(3):e0248707. doi: 10.1371/journal.pone.0248707 (PMC7971505; doi:10.1371/journal.pone.0248707)
Supplement: Attachment — Submitted filename: Comments on PLoS One article.docx [file pone.0248707.s010.docx]

"Drug-resistant TB prevalence study in 5 health institutions in Haiti"

I congratulate the authors who have brought data on DR-TB in Haiti pertaining to patients and mycobacterial characteristics under difficult circumstances of strike.

From this manuscript it is evident that the authors wanted to conduct this study in five sites across Haiti by enrolling 1000 new and 250 re-treatment TB cases and to characterise the DR-TB isolates further. However, due to strike in the peripheral regions, the study was mainly conducted in the central region, IMIS and INLR (contributing 2504 enrolments) as compared to peripheral regions (273 enrolments) from April 2016 to February 2018. Out of these, 2401 were new cases while 376 were re-treatment cases. I wonder if the samples/patients from the peripheral sites (HIC, HUJ and HUM) were referred to central laboratories for diagnosis to make the title relevant as DR-TB prevalence in regions catered by 5 health institutions.

Minor Comments:

Abstract:

1. Objectives: Please write the objectives of the study clearly.
2. Results: The authors may give their own results only, avoiding comparison with the previous studies that may be done in the discussion part of the main manuscript.
3. Conclusions: The conclusions can be crisp and corroborate the objectives.

Manuscript:

1. Introduction: The part “including about 8400 to 10000 adolescents ------retention in care” may be avoided.
2. Line 59, MDR-TB infections may replaced with MDR-TB disease
3. The case management of such patients is costly for local healthcare systems and threatens to negatively impact of the progress made in the recent years in the fight against TB in Haiti. “of” may be deleted.
4. Line 94 to 95: Exclusion criteria included age under 15, negative smear microscopy, and signs of extrapulmonary TB. This sentence does not match with lines 109 to 110 where it is mentioned “The protocol conducted by the GHESKIO centers (INLR and IMIS) was slightly different as only two sputum specimens were collected per patient, and no microscopy testing was performed”. These two sites were main contributors for the study.
5. Line 136-137: How was sample size of 1250 arrived at? And why did the authors exceed the pre-determined sample size? Do 2777 cases represent all the cases during the study duration that satisfy the inclusion criteria or some other sampling techniques was used considering the strike in the regions.
6. Line 141: unequal between sites may be replaced with unequal among sites.
7. Line 154-156: Please clarify if the study had 62 isolates that were MDR among total 74 DR TB cases and also 62 DR TB isolates that were identified in new TB cases. Please also clarify the type of resistance found in new and re-treatment cases.
8. Table 2: In treatment after interruption, how was p value of 0.118 obtained when there was no DR-TB case?
9. Line 175-184: This part may be shifted to discussion.
10. While you use the word “cohort” in discussion in lines 225, 231 and 237, what do we understand? Can were replace it with “the tested isolates”
11. Line 269, can we make it “detection rate of HIV-TB co-infection”?
12. Conclusions: Please make the conclusions in-line with the objectives.
13. References: Many of the references are in French.
14. Supplementary material Table 1S: In the heading, Treatment column is depicted twice, please make it as Treatment after interruption and Treatment after failure.
15. Table 1S: Resistant to 3AB* - the line total should be 13.
16. Figure 1S: If you give number of isolates also, it will clarify the confusion of MDR-TB cases among total DR-TB cases.
